# Nursing: Forever Changed by a Pandemic

**DOI:** 10.1177/0894318420943134

**Published:** 2020-10

**Authors:** Pamela N. Clarke

**Affiliations:** 1 University of Wyoming, Laramie, WY, USA

**Keywords:** coronavirus, moral injustice, nursing knowledge, nursing practice, pandemic

## Abstract

The paper consists of reflections on the corona pandemic and nursing knowledge as practice. Nurses have been so appreciated by the public during this time that they are referred to as heroes. The moral injustice of taking nurses who come so willing to serve and not provide protective gear for their practice is addressed.

As 2020 began to evolve, people on the planet were going about their usual activities, when the world suddenly stopped due to a highly contagious germ. The pandemic was announced and people everywhere moved to the safety of their homes and ostensibly waited for the pandemic to run its course or for some indication of what the next steps should be. The initial shock was so great that people were slow to understand the implications for themselves and the public. Was it that serious? The numbers began to rise and there was a realization that it was worldwide. As many people settled into their homes to be safe from the virus, critical care nurses ran to care for the sick and dying. Additional noncritical care nurses prepared through the American Nurses Association (ANA) webinars were encouraged to help with critical care for those on ventilators. Having attended one of those webinars, I became acutely aware of how desperate the situation was. The line of volunteer critical care nurse providers was going to be woefully inadequate and minimally prepared.

The stories of nurses and other providers caring for those who are dying are both inspiring and heartbreaking. The lives of those who care suffer significant impact on their emotions and their selves as human beings. Carers are both touched and traumatized by the experiences of those who survived and those who did not. Nurses across the country shared poignant moments where patients knew that the ventilator represented death, yet they had come to connect the person to a vent, knowing the person couldn’t even see their faces. Nurses shared how they struggled with a room full of ventilator patients unable to speak; the ever-present sound of the machines that were keeping people alive expressed as a deafening sound of high-pressure air together with constant reminders from the alarms.

Public appreciation for nursing care resounded around the world. In a paradox, citizens were expressing their gratitude for quality nursing care, and hospital administrators were embedded in moral outrage as they were struggling to get protection for nurses so they could continue to care for C-19 patients. It is a fact that the public trusts nurses more than any other profession and have for the past 18 years. The most recent survey (Reinhart, 2020) confirmed that nurses are again the number one most trusted profession. In the war-like scenario, public respect for the disciplinary knowledge, skills, and humanity of nurses were on display like never before. The care was exceptional and it was clear that patients were receiving state-of-the-art acute and critical care keeping people alive. Reading and hearing the stories of nurses and patients can be devastatingly heartbreaking yet at the same time recognizing the immense hero factor attributed to nurses and feeling incredibly proud to be a nurse. While the public recognized nurses as heroes, their vulnerability soon became apparent as the critical numbers faded through illness and death. In the patient care arena, it has not been easy. Not unexpectedly many devoted nurses and physicians have become ill and died, just like the patients they were caring for in all age groups. Communities grieved and, in some cases, memorialized their caregivers. The impact of public approval on the population of nurses is significant as nurses are being recognized as key providers all over the world. In some areas of the world, citizens sang praises to nursing staff as they were coming to serve the hospital and as they were coming home. Citizens felt safer, knowing there were nurses to care for them.

The safety of the nurses became an issue all over the United States due to shortages of equipment. Obtaining effective masks and personal protective equipment (PPE) was paramount for the hospitals so that they could keep taking patients. Unbelievably, it seemed little help was coming from the federal stockpiles, and even more shocking, it became clear that what was in the stockpiles was insufficient. Stories of the Federal government stealing from states (whether true or not) were rampant and challenging for healthcare workers and the public to comprehend. As a public health nurse watching now, instead of directing and planning, feelings of desperation and outrage were primary as the system seemed to fail the hospitals, the patients, and frontline workers. Staff members in some areas, such as nursing homes, were faced with treating patients with no protection and putting their lives at risk or not treating them at all. Reported lack of equipment and supplies were in such vast numbers (40,000 ventilators) that obtaining sufficient amounts was incomprehensible. Distress over huge numbers of deaths in nursing homes, what to do with the dead bodies, conflict over what to do with patients who are no longer critical to make room for the influx of new patients, and waiting for testing and contact tracing were taking a toll on the workforce and the public health systems.

Nursing knowledge continues to be a force needed in every component of the epidemic, especially in areas where vulnerable populations are dying in such large numbers. Planning and creating different systems using nursing models is in short supply. Nursing homes have been continuing the same mode of operations instead of implementing true infection control to quell the pace of death.

After 2 months of “sheltering in place,” the world is ready to open. The epidemiologist report by Dr. Anthony Fauci to the United States Senate made it clear that the reopening of the public spaces should be done very slowly (many months) with extensive testing and contact tracing available (Montanaro, 2020). As a public health nurse, I picture this highly contagious virus invading public spaces as people emerge from their homes and travel to vacation spots or even just go out to dinner or meet up with friends. Since Dr. Fauci is clearly not in charge of the “reopening,” the expected outcome is likely to be more cases and chaos in some places.

When I reflect on nursing theory, it is personified in those men and women serving the C-19 patients on the line and nurses in administration keeping the system going. The public and health professionals have been confident in the state of nursing science in the workforce, as employees and hundreds of volunteers began their journey to tackle the pandemic. Yet the disease did not succumb to the best care and national public isolation. Two months after the initial decisions to close public services and “shelter at home,” the number of cases is still growing at exponential rates, in some areas, along with a worrisome developing plan to open businesses throughout the country. Faculty members are struggling with decisions about how to keep students safe in the upcoming semester, now that the universities are making decisions to bring students back to campus.

The uncertainty surrounding the disease and what seems to be the changing symptoms have some people wondering if C-19 is evolving into a different condition. The future is not known. Will the immunity that one acquires hold for the emerging differences over the long term? The concept of immunity is still being questioned, and as yet, there is no clear assurance that herd immunity is achievable.
